# Epilepsy and EEG abnormalities in neurodegenerative dementias: toward a system epilepsy framework

**DOI:** 10.3389/fnagi.2026.1806471

**Published:** 2026-03-31

**Authors:** Paolo Manganotti, Federica Palacino, Stefania Pavan, Alberto Benussi

**Affiliations:** 1 Neurology Unit, Department of Medical, Surgical and Health Sciences, University of Trieste, Trieste, Italy; 2 Neurology Unit, Hospital Care Department of Medicine, Azienda Sanitaria Universitaria Giuliano Isontina, Trieste, Italy

**Keywords:** Alzheimer’s disease, dementia with Lewy bodies, electroencephalography, epilepsy, frontotemporal dementia, network hyperexcitability, subclinical epileptiform activity, system epilepsy

## Abstract

Epilepsy and epileptiform activity represent underrecognized yet clinically significant features of neurodegenerative dementias, with emerging evidence suggesting they may contribute to disease progression rather than merely representing epiphenomena of neuronal loss. This comprehensive review examines the epidemiology, clinical presentation, electroencephalographic findings, and pathophysiological mechanisms underlying seizure activity in Alzheimer’s disease (AD), dementia with Lewy bodies (DLB), and frontotemporal dementia (FTD). Meta-analytic data demonstrate elevated seizure prevalence across all three conditions, with cumulative probabilities of 13.4% for AD, 14.7% for DLB, and 3.0% for FTD, representing risk elevations of approximately 6- to 10-fold compared to age-matched controls. Critically, subclinical epileptiform activity detected through prolonged electroencephalographic monitoring affects up to 42-54% of AD patients and is associated with 1.5-fold accelerated cognitive decline. Each dementia subtype exhibits characteristic electroencephalographic signatures: AD demonstrates progressive spectral slowing with predominantly left temporal epileptiform discharges; DLB shows highly characteristic slowing of the dominant rhythm below 8 Hz with high diagnostic accuracy; and FTD displays relatively preserved background activity with frontal-temporal hypoconnectivity. We synthesize evidence from transcranial magnetic stimulation studies demonstrating distinct patterns of cortical excitability alterations across these conditions, with AD showing bilateral increases in cortical excitability and reduced GABAergic and cholinergic inhibition. Building upon these observations, dementia-associated epilepsy may be conceptualized within the framework of system epilepsies, arising from dysfunction of vulnerable neural networks rather than discrete lesions. This paradigm shift has profound therapeutic implications, supporting network-targeted interventions and the potential disease-modifying role of antiseizure medications. We conclude by presenting clinical recommendations for monitoring and treatment, emphasizing the need for prolonged electroencephalographic evaluation and consideration of empirical treatment for subclinical epileptiform activity associated with cognitive fluctuations.

## 1 Introduction

The relationship between epilepsy and neurodegenerative dementias has gained increasing scientific attention over the past two decades, fundamentally reshaping our understanding of both conditions (Vossel et al., 2017a; Cretin, 2018; Sen et al., 2018). What was once considered a rare, late-stage complication of dementia is now recognized as a prevalent phenomenon that may occur early in the disease course and potentially contribute to cognitive decline (Palop and Mucke, 2016; Vossel et al., 2021). This paradigm shift carries profound implications for clinical practice and therapeutic development.

Epilepsy incidence demonstrates a bimodal distribution across the lifespan, with peaks in childhood and late adulthood (Hauser et al., 1993; Fiest et al., 2017). Landmark epidemiological studies in the United States have established that epilepsy incidence rises dramatically with age, increasing from 28 cases per 100,000 individuals per year at age 50 to 139 per 100,000 at age 70 (Hauser et al., 1993). Finnish cohort data reveal a nearly fivefold increase in epilepsy incidence among individuals aged 60 years and older between 1973 and 2013, largely attributable to population aging (Sillanpää et al., 2016). Within this elderly population, neurodegenerative diseases account for approximately 10% of new-onset epilepsy cases, with Alzheimer’s disease (AD) representing the dementia subtype most frequently associated with seizures (Mendez and Lim, 2003; Vu et al., 2018).

The clinical significance of epileptic activity in dementia extends beyond the immediate morbidity associated with seizures. Converging evidence from both human studies and animal models suggests a bidirectional relationship between epilepsy and neurodegeneration: dementia predisposes to epileptic activity, and epileptic activity, in turn, may accelerate cognitive decline and disease progression (Noebels, 2011; Lam et al., 2017; Vossel et al., 2017a). This hypothesis is supported by observations that patients with AD who develop epilepsy experience earlier symptom onset and more rapid cognitive deterioration (Volicer et al., 1995; Vossel et al., 2013a), and that subclinical epileptiform activity correlates with faster decline on standardized cognitive assessments (Lam et al., 2020; Horvath et al., 2021).

Electroencephalography (EEG) has emerged as a critical tool for understanding neural network dysfunction in dementia (Babiloni et al., 2020; Rossini et al., 2020a). Beyond its traditional role in seizure detection, quantitative EEG analysis provides insights into the spectral and connectivity alterations that characterize different dementia subtypes (Jeong, 2004; Dauwels et al., 2010). Each neurodegenerative condition exhibits relatively distinct EEG signatures, reflecting the topographical distribution and molecular pathology of the underlying disease process (Bonanni et al., 2008; Nardone et al., 2011). These findings have practical diagnostic applications, particularly for differentiating dementia with Lewy bodies (DLB) from AD, where EEG demonstrates high discriminative accuracy (Bonanni et al., 2015; McKeith et al., 2017).

The pathophysiological mechanisms linking neurodegeneration to epileptogenesis involve complex interactions between pathological protein aggregates and neural network function (Palop et al., 2007; Zott et al., 2019). Amyloid-beta oligomers, tau hyperphosphorylation, alpha-synuclein aggregation, and TDP-43 pathology have each been implicated in promoting neuronal hyperexcitability through distinct but overlapping mechanisms, including disruption of GABAergic inhibition, enhancement of glutamatergic excitation, and destabilization of network oscillations (Sanchez et al., 2012; Busche and Konnerth, 2016; Palop and Mucke, 2016). Transcranial magnetic stimulation (TMS) studies provide complementary evidence of altered cortical excitability across dementia subtypes, revealing reduced intracortical inhibition and enhanced facilitation in AD and related conditions (Di Lazzaro et al., 2002; Benussi et al., 2021a; Benussi and Vucic, 2026).

In this comprehensive review, we argue that epilepsy associated with neurodegenerative dementias may be conceptualized within the framework of system epilepsies. Originally described in the context of childhood epileptic syndromes such as self-limited epilepsy with centrotemporal spikes (Rolandic epilepsy), the system epilepsy concept emphasizes seizure generation from functionally characterized brain networks rather than discrete structural lesions (Capovilla et al., 2009; Avanzini et al., 2012). We argue that the bilateral cortical excitability changes, network-level pathophysiology, and absence of circumscribed epileptogenic zones characteristic of dementia-associated epilepsy align more closely with this framework than with traditional concepts of focal symptomatic epilepsy.

Growing evidence from studies on late-onset epilepsy of unknown origin further supports a network-based perspective in aging-related epileptogenesis. Older adults with new-onset seizures in the absence of overt structural lesions show an increased risk of cognitive impairment and subsequent dementia, suggesting that epileptogenesis may precede or parallel neurodegenerative processes (Reyes et al., 2023). Meta-analyses and prospective studies indicate that late-onset unexplained epilepsy is associated with distinct cognitive phenotypes and a higher likelihood of progression to mild cognitive impairment or dementia (Sen et al., 2018; Tang et al., 2022; Reyes et al., 2023). These findings challenge a purely lesion-centered model and instead point toward shared mechanisms of network vulnerability, including synaptic dysfunction, neuroinflammation, and system-level instability in aging brains.

This review synthesizes current evidence on epilepsy and EEG abnormalities across three major neurodegenerative dementias: AD, DLB, and frontotemporal dementia (FTD). We examine epidemiological data, clinical presentations, EEG findings, pathophysiological mechanisms, and therapeutic implications. Our objectives are threefold: first, to provide clinicians with a comprehensive understanding of seizure risk and EEG monitoring strategies across dementia subtypes; second, to articulate the system epilepsy hypothesis as a unifying framework for understanding dementia-associated epilepsy; and third, to highlight the potential for antiseizure medications to serve as disease-modifying agents through their effects on network hyperexcitability.

## 2 Epidemiology of epilepsy in neurodegenerative dementias

Accurate epidemiological characterization of epilepsy in neurodegenerative dementias presents substantial methodological challenges (Friedman et al., 2012; Subota et al., 2017). Studies vary widely in their ascertainment methods, ranging from retrospective medical record review to prospective surveillance with extended EEG monitoring. Definitions of epilepsy itself differ, with some studies including isolated seizures while others require recurrent unprovoked seizures meeting International League Against Epilepsy (ILAE) criteria (Fisher et al., 2014). The severity of dementia in study populations, availability of histopathological confirmation, and length of follow-up further contribute to heterogeneity in reported estimates (Pandis and Scarmeas, 2012). Despite these limitations, meta-analytic approaches have begun to establish reasonably consistent prevalence and incidence figures across dementia subtypes (Zelano et al., 2020; Zhao et al., 2021; see Table 1).

**Table 1:** Epidemiology of epilepsy across neurodegenerative dementias.

Parameter	Alzheimer’s disease	Dementia with Lewy bodies	Frontotemporal dementia
Point prevalence	3.0-6.4%	2.6-4.7%	2.0-11%
Cumulative probability	13.4%	14.7%	3.0%
Risk vs. controls	~10-fold	~10-fold	~6-fold
Myoclonus (cumulative)	42%	58%	Rare
Peak risk period	Advanced stages; early-onset AD	Throughout disease	5 + years post-diagnosis

AD, Alzheimer’s disease.

### 2.1 Alzheimer’s disease

AD represents the dementia subtype most extensively studied with respect to epilepsy comorbidity (Vossel et al., 2017a, 2026; Asadollahi et al., 2019). A comprehensive systematic review and meta-analysis identified a pooled seizure prevalence of 4.86% (95% CI: 3.43–6.51%) across AD populations (Zhao et al., 2021). However, this figure likely underestimates true prevalence given the high proportion of subclinical seizures and the insensitivity of routine clinical ascertainment (Vossel et al., 2016a). Cumulative probability estimates, which account for the progressive nature of both conditions, suggest that approximately 13.4% of AD patients will experience seizures over the disease course (Beagle et al., 2017).

The risk of epilepsy in AD relative to the general elderly population has been quantified across multiple cohort studies (Amatniek et al., 2006; Scarmeas et al., 2009; Irizarry et al., 2012; Palacino et al., 2025b). Estimates range from 2.8-fold (Lyou et al., 2018) to 10-fold (Amatniek et al., 2006), with variation reflecting differences in control group selection and ascertainment intensity. A large cohort study utilizing the Korean National Health Insurance Service-Senior Cohort Database demonstrated that AD patients were 2.8 times more likely to develop epilepsy than age-matched controls, with new-onset epilepsy associated with a 1.5-fold increase in mortality risk (Lyou et al., 2018). Other population-based studies have confirmed substantially elevated risk ratios ranging from 5.3 to 10-fold (Hesdorffer et al., 1996; Sherzai et al., 2014).

Age at dementia onset represents a critical modifier of seizure risk (Mendez et al., 1994; Lozsadi and Larner, 2006). Early-onset AD (onset before age 65) confers particularly elevated risk, with hazard ratios of 4.06 (95% CI: 3.25-5.08) for developing epilepsy within 5 years compared to age-matched controls (Zarea et al., 2016). Among individuals under age 50 with AD, the risk may be elevated 87-fold relative to the general population (Amatniek et al., 2006). This association is particularly pronounced in familial AD associated with pathogenic mutations in *APP*, *PSEN1*, or *PSEN2*, where seizure prevalence ranges from 30 to 50% depending on the specific mutation (Snider et al., 2005; Larner and Doran, 2006; Pandis and Scarmeas, 2012).

Myoclonus, distinct from epileptic seizures but sharing pathophysiological overlap, occurs in 7-10% of AD patients overall, with cumulative risk reaching 80% in advanced disease stages (Hauser et al., 1986; Chen et al., 1991). The presence of myoclonus confers nearly eight-fold increased risk of subsequent seizures (Beagle et al., 2017). In atypical AD variants with prominent neocortical involvement, such as posterior cortical atrophy and logopenic variant primary progressive aphasia, myoclonus prevalence may exceed 30% (Caviness, 2003).

### 2.2 Dementia with Lewy bodies

DLB, the second most common neurodegenerative dementia, has received comparatively less attention regarding epilepsy comorbidity (Walker et al., 2015; McKeith et al., 2017). Available data suggest seizure prevalence of 2.68% (95% CI: 2.13–3.28%) based on meta-analytic estimates (Zhao et al., 2021). However, cumulative probability data reveal a lifetime seizure risk of 14.7%, comparable to or exceeding that observed in AD (Beagle et al., 2017). This apparent discrepancy between point prevalence and cumulative probability may reflect differences in disease duration, with DLB typically following a more rapid clinical course than AD (Williams et al., 2006).

The relative risk of epilepsy in DLB compared to controls parallels that observed in AD, with approximately 10-fold elevation (Beagle et al., 2017). Notably, DLB demonstrates the highest cumulative incidence of myoclonus among the major neurodegenerative dementias, reaching 58% over the disease course (Beagle et al., 2017). The high frequency of myoclonus in DLB may relate to the prominent involvement of brainstem and cortical motor circuits by alpha-synuclein pathology (Caviness et al., 2010).

An important consideration in interpreting DLB epidemiological data is the frequent co-occurrence of AD pathology (Toledo et al., 2013; Irwin et al., 2017). Autopsy studies reveal that 86% of clinically diagnosed DLB cases demonstrate significant neuritic plaques and/or tauopathy meeting neuropathological criteria for concomitant AD (Irwin et al., 2017). This mixed pathology likely contributes to epileptogenesis through both alpha-synuclein and amyloid/tau-mediated mechanisms, potentially explaining the comparable seizure risk between pure AD and DLB populations (Vicente et al., 2024).

### 2.3 Frontotemporal dementia

FTD encompasses a heterogeneous group of disorders characterized by frontal and/or temporal lobe degeneration, with three main clinical variants: behavioral variant FTD (bvFTD), semantic variant primary progressive aphasia, and nonfluent/agrammatic variant primary progressive aphasia (Gorno-Tempini et al., 2011; Rascovsky et al., 2011; Benussi et al., 2022a). Underlying neuropathology is similarly heterogeneous, including tau-predominant forms (Pick’s disease, progressive supranuclear palsy, corticobasal degeneration), TDP-43 proteinopathies, and FUS proteinopathies (Mackenzie and Neumann, 2016; Borroni and Benussi, 2019; Palacino et al., 2025a).

Meta-analytic seizure prevalence in FTD ranges from 2.0 to 2.81% (Zhao et al., 2021), with cumulative probability of 3.0% (Beagle et al., 2017). These figures are notably lower than those for AD or DLB, representing approximately 6-fold elevation relative to age-matched controls rather than the 10-fold elevation observed in amyloidopathies (Beagle et al., 2017). The lower seizure risk in FTD may reflect the relative absence of amyloid-beta pathology in most FTD subtypes, consistent with mechanistic evidence implicating amyloid oligomers as particularly epileptogenic (Palop et al., 2007; Sanchez et al., 2012).

Recent Finnish cohort data (Kilpeläinen et al., 2025) provide important longitudinal perspective on the temporal relationship between epilepsy and FTD. Remarkably, epilepsy prevalence was already elevated to 3.3% versus 0.8% in controls at 10 years before dementia diagnosis, suggesting that shared pathophysiological mechanisms may be active long before clinical dementia onset. By 5 years post-diagnosis, prevalence reached 11%. Behavioral variant FTD accounted for 54-58% of FTD-epilepsy cases (Kilpeläinen et al., 2025).

Within FTD, seizure risk appears to vary by underlying pathology and genetic etiology (Snowden et al., 2015; Van Mossevelde et al., 2018). Studies examining *C9orf72* mutation carriers, the most common genetic cause of familial FTD, have not demonstrated significantly increased seizure risk compared to non-carriers with FTD (Devenney et al., 2018).

## 3 Seizure semiology and clinical presentation

The clinical presentation of seizures varies substantially across dementia subtypes, reflecting differences in the anatomical distribution of pathology and the functional specialization of involved neural networks (Vossel et al., 2017a; Baker et al., 2019). Accurate characterization of seizure semiology is critical for diagnosis, as many seizure manifestations in dementia patients may be subtle and easily attributed to dementia-related symptoms rather than recognized as epileptic phenomena (Rao et al., 2009; Cretin et al., 2016).

### 3.1 Seizure types in Alzheimer’s disease

In AD, focal seizures with impaired awareness predominate, accounting for 70-72% of epileptic events (Mendez and Lim, 2003; Horváth et al., 2018). These seizures typically arise from mesial temporal structures, consistent with the early and prominent involvement of hippocampal and entorhinal cortex in AD pathology (Braak and Braak, 1991). Left temporal predominance is observed in both ictal semiology and interictal epileptiform discharges, possibly reflecting the language-dominant hemisphere’s vulnerability or ascertainment bias related to verbal symptom reporting (Vossel et al., 2016a).

A striking feature of AD-associated epilepsy is the high proportion of non-motor seizures. Horváth et al. (2018) found that 55% of focal seizures in AD lacked motor manifestations, presenting instead with experiential phenomena, behavioral arrest, or subtle alterations in cognition. These non-motor manifestations include *déjà vu* and *jamais vu* experiences, sensory phenomena (metallic taste, burning odor, ascending epigastric sensations, and thoracic warmth), psychic phenomena (intense fear or sudden apathy), speech arrest, transient aphasia, and episodic amnesia (Vossel et al., 2013a; Cretin et al., 2016). Such symptoms are frequently attributed to dementia-related fluctuations rather than recognized as seizures, contributing to underdiagnosis (Baker et al., 2019).

Transient epileptic amnesia (TEA) represents a particularly relevant seizure type in the context of AD (Zeman et al., 1998; Butler and Zeman, 2008). TEA manifests as recurrent episodes of isolated amnesia lasting minutes to hours, typically occurring upon awakening, with preserved consciousness during events. Patients may express mild concern about memory lapses while otherwise appearing normal. The amnesia may be anterograde, retrograde, or both, with gradual but sometimes incomplete resolution (Butler et al., 2007). Diagnostic criteria require a history of recurrent transient amnesia with cognitive function preserved between episodes, accompanied by evidence supporting epilepsy diagnosis such as EEG epileptiform discharges, other seizure types, or response to antiseizure medication (Butler and Zeman, 2008).

TEA has been proposed as a potential mechanism underlying wandering episodes in AD patients based on case reports and small series (Rabinowicz et al., 2000; Palop and Mucke, 2009; Cretin et al., 2023). Case reports describe dementia patients experiencing recurrent transient episodes of amnesic wandering and disorientation, even in familiar environments, who subsequently demonstrated interictal epileptiform discharges on EEG (Del Felice et al., 2014). While the contribution of TEA to wandering behavior in AD requires further investigation, this association highlights the importance of considering epileptic etiologies for behavioral disturbances in dementia.

Generalized tonic–clonic seizures occur less frequently in AD, typically representing secondary generalization from focal onset (Rao et al., 2009; Pandis and Scarmeas, 2012). These seizures are more common in advanced disease stages and may be the seizure type most readily recognized by caregivers and clinicians. However, their frequency in AD does not markedly exceed that observed in the healthy elderly population, in contrast to the substantially elevated rates of focal seizures (Vossel et al., 2013a).

### 3.2 Seizure types in dementia with Lewy bodies

DLB demonstrates a somewhat different seizure profile than AD, with a higher proportion of generalized seizures (75%) relative to focal seizures (Rao et al., 2009; Beagle et al., 2017). This pattern may reflect more diffuse cortical involvement by alpha-synuclein pathology or the frequent co-occurrence of AD pathology with its associated epileptogenic mechanisms (Irwin et al., 2017).

Diagnostic challenges in DLB are compounded by the core clinical features of the disease itself (McKeith et al., 2017). Cognitive fluctuations, a hallmark of DLB, may closely resemble postictal confusion or nonconvulsive seizure states (Ferman et al., 2004). Visual hallucinations, another core feature, must be differentiated from ictal visual phenomena (Taylor et al., 2011). Parkinsonism may mask or modify the motor manifestations of seizures. REM sleep behavior disorder, present in the majority of DLB patients, involves complex motor behaviors during sleep that could theoretically be confused with nocturnal seizures, though the clinical context typically permits differentiation (Boeve et al., 2007).

Myoclonus is particularly prevalent in DLB, reaching 58% cumulative incidence (Beagle et al., 2017). While myoclonus is not synonymous with epilepsy, it reflects cortical hyperexcitability and frequently co-occurs with epileptic seizures (Manganotti et al., 2006; Caviness et al., 2010). The presence of myoclonus in DLB may indicate shared pathophysiological mechanisms involving disrupted cortical inhibition and should prompt consideration of EEG monitoring (Caviness, 2003).

### 3.3 Seizure types in frontotemporal dementia

FTD-associated seizures are predominantly focal (42-50%), with temporal lobe epilepsy representing the most common focal subtype (36-42% of cases; Beagle et al., 2017; Kilpeläinen et al., 2025). This temporal predominance is somewhat surprising given the classical frontotemporal distribution of pathology but may reflect the relatively greater epileptogenicity of temporal lobe structures compared to frontal regions (Engel, 2001).

An important clinical observation is the favorable response to antiseizure medications in FTD-associated epilepsy (Beagle et al., 2017). Studies report seizure freedom rates of 87.5% with pharmacotherapy, substantially exceeding the 50-70% seizure freedom typically achieved in general epilepsy populations (Kwan and Brodie, 2000). This favorable treatment response may reflect different epileptogenic mechanisms in FTD compared to AD, or alternatively may represent selection bias if milder FTD cases are more likely to survive long enough to develop and receive treatment for epilepsy.

### 3.4 Seizure types in other dementia subtypes

Parkinson’s disease dementia may show diffuse slowing but rarely epileptiform activity; subclinical seizures are uncommon (Musaeus et al., 2023). Limbic-predominant age-related TDP-43 encephalopathy (LATE) and primary age-related tauopathy (PART) are recently defined neuropathological entities diagnosed at autopsy, and dedicated EEG or epileptiform activity data remain lacking. Sporadic Creutzfeldt-Jakob disease (sCJD) is characterized by periodic sharp-wave complexes (PSWCs), typically occurring at approximately 0.5–2 Hz, although these may be absent in certain molecular subtypes (e.g., MM2-cortical; Hermann et al., 2021). Vascular dementia shows decreased alpha power, increased delta and theta power, and altered connectivity, but findings are heterogeneous and lack specificity. Expert panels recommend EEG as a research tool for understanding neural synchronization and the effects of cerebrovascular lesions (Babiloni et al., 2021).

### 3.5 Subclinical seizures: the hidden burden

Perhaps the most significant recent advance in understanding epilepsy in dementia has been the recognition that subclinical epileptiform activity affects a substantial proportion of patients and carries prognostic significance (Vossel et al., 2016a; Lam et al., 2017). Vossel et al. (2016a) demonstrated subclinical epileptiform activity in 42.4% of AD patients using combined EEG and magnetoencephalography (MEG) monitoring. With 24-h EEG, detection rates range from 22 to 54%, compared to only 2-3% with routine 20–30-min EEG recordings (Brunetti et al., 2020; Lam et al., 2020).

This subclinical activity occurs predominantly during slow-wave sleep, explaining why daytime recordings miss the majority of events (Lam et al., 2017; Horváth et al., 2018). Silent hippocampal seizures, demonstrated via *foramen ovale* electrode recordings by Lam et al. (2017), revealed that 90-100% of hippocampal spikes remain invisible to scalp recordings, further emphasizing the limitations of conventional EEG monitoring.

Emerging longitudinal evidence strongly suggests that subclinical epileptiform activity is clinically meaningful in AD, though interventional trials demonstrating improved outcomes with treatment are still awaited (Lam et al., 2020; Horvath et al., 2021). Horvath et al. (2021) followed AD patients longitudinally and found that those with subclinical epileptiform activity experienced 1.5-fold faster cognitive decline compared to those without. Annual Mini-Mental State Examination (MMSE) decline was 3.9 points with subclinical activity versus 1.6 points without (Vossel et al., 2016a). Spike frequency correlated strongly with decline rate, suggesting a dose–response relationship between epileptiform burden and cognitive deterioration (Horvath et al., 2021). These findings position subclinical epileptiform activity as both a biomarker and potential therapeutic target in AD.

## 4 Electroencephalographic findings in neurodegenerative dementias

Electroencephalography provides a window into the neural network dysfunction underlying neurodegenerative dementias, revealing both the characteristic spectral alterations that accompany cognitive decline and the epileptiform abnormalities that may contribute to disease progression (Dauwels et al., 2010; Rossini et al., 2020a). Each dementia subtype demonstrates relatively distinct EEG signatures, reflecting differences in the anatomical distribution, molecular pathology, and neurotransmitter system involvement characteristic of each condition (Jeong, 2004; Babiloni et al., 2020; see Table 2). The strength of evidence supporting EEG use varies across dementia subtypes (De Keulenaer et al., 2025). In AD, EEG is not routinely indicated for diagnosis but is increasingly explored as a biomarker, particularly through quantitative approaches; however, limited specificity and lack of standardization currently restrict its clinical implementation (Rossini et al., 2020b). In DLB, EEG is a well-established supportive diagnostic biomarker and is frequently used in both clinical and research settings, with robust evidence for characteristic diffuse slowing and good discriminative value from AD (Zarkali et al., 2025). In FTD, evidence remains limited and heterogeneous, and routine EEG use is not currently supported (De Keulenaer et al., 2025). Although certain electroclinical patterns have been described across syndromes, no reproducible EEG subtypes can be defined, as most abnormalities are diffuse and bilateral, consistent with widespread network dysfunction.

**Table 2:** Characteristic EEG features across neurodegenerative dementias.

Feature	Alzheimer’s disease	Dementia with Lewy bodies	Frontotemporal dementia
Dominant rhythm	Progressive slowing; preserved reactivity early	Slowed <8 Hz (90%)	Relatively preserved
Spectral changes	Increased theta/delta; decreased alpha/beta	Marked slowing; FIRDA	Decreased frontal alpha/beta; preserved fast frequencies
IED localization	Left temporal (57%); bitemporal (26%)	Less well characterized	Temporal predominance
IED prevalence (24 h EEG)	22-54%	Less studied	Less studied

EEG, electroencephalography; Hz, Hertz; FIRDA, frontal intermittent rhythmic delta activity; IED, interictal epileptiform discharge.

### 4.1 EEG characteristics in Alzheimer’s disease

The EEG changes in AD follow a predictable pattern that correlates with disease severity and reflects progressive disruption of thalamocortical and corticocortical networks (Prichep, 2007; Babiloni et al., 2016b; Palacino et al., 2025b). The most consistently observed abnormality is slowing of the posterior dominant rhythm, which normally oscillates in the alpha frequency range (8-13 Hz) in healthy adults (Huang et al., 2000). In mild cognitive impairment (MCI) and early AD, the dominant rhythm may remain within the alpha range but demonstrate reduced reactivity to eye opening and diminished amplitude (Babiloni et al., 2004). As disease progresses, the dominant rhythm slows into the theta range (4-8 Hz) and eventually the delta range (< 4 Hz) in advanced stages (Coben et al., 1985; Penttilä et al., 1985).

Quantitative EEG (qEEG) analysis provides more sensitive detection of AD-related changes than visual inspection alone (Jelic et al., 2000; Prichep et al., 2006). Spectral analysis consistently demonstrates increased delta and theta power, decreased alpha and beta power, and increased theta-to-alpha ratio in AD relative to age-matched controls (Rossini et al., 2020a; Babiloni et al., 2025; Benussi et al., 2026). These changes show regional specificity, with slowing most pronounced in temporal and parietal regions corresponding to areas of greatest AD pathology and hypometabolism on PET imaging (Dierks et al., 2000; Babiloni et al., 2020). The alpha-to-delta power ratio and theta/gamma ratio have been proposed as potential diagnostic and prognostic biomarkers (Jelic et al., 2000; Gallego-Jutglà et al., 2014; Bonanni et al., 2021b; Bonanni et al., 2021a; Lopez et al., 2023).

Specific qEEG markers have demonstrated utility in predicting conversion from MCI to AD (Jelic et al., 2000; Moretti et al., 2008; Rossini et al., 2020a). Across these studies, MCI was diagnosed according to established clinical criteria (largely Petersen-derived), requiring subjective cognitive complaints, objective impairment on standardized neuropsychological testing, preserved activities of daily living, and absence of dementia. Global cognition and staging were assessed using instruments such as the Mini-Mental State Examination (MMSE) and Clinical Dementia Rating (CDR), with systematic medical, neurological, and MRI evaluation to exclude alternative causes. In studies involving AD patients, diagnoses were established according to NINCDS-ADRDA criteria and, in more recent cohorts, confirmed within biomarker-supported frameworks (IWG/NIA-AA) including structural MRI and FDG-PET. In these clinically well-characterized samples, increased fast alpha activity (alpha3), elevated alpha3/alpha2 ratio, and increased theta/gamma ratio have each been associated with subsequent conversion (Moretti et al., 2011). The alpha3/alpha2 ratio correlates with temporoparietal cortical thinning and memory impairment, and its combination with cortical thickness measurement may identify individuals at elevated risk for progression to dementia (Babiloni et al., 2016a). High alpha3/alpha2 ratios predict poorer performance on verbal learning tests and are associated with reduced hippocampal volumes (Moretti et al., 2008).

Coherence and connectivity analyses reveal disrupted functional integration in AD (Babiloni et al., 2004; Stam et al., 2005). Interhemispheric coherence is reduced, particularly in alpha and beta frequency bands, reflecting the corticocortical disconnection that characterizes AD pathophysiology (Knott et al., 2000). Decreased alpha coherence correlates with neuropsychological test performance and has been proposed as a marker of quality of life in AD patients (Fonseca et al., 2015). Phase synchronization in the theta band shows delayed patterns that correlate with MMSE scores (Koenig et al., 2005).

EEG abnormalities correlate with AD biomarkers (Kramberger et al., 2013a; Smailovic et al., 2018). Subjects with high total tau and low amyloid-beta_1-42_ in cerebrospinal fluid demonstrate increased theta power and enhanced slow-wave activity regardless of clinical diagnosis (Kramberger et al., 2013b; Tsolaki et al., 2014). The DIMENSION method (Neuronal Dysfunction Method) identified negative correlations between alpha dipolarity and both phosphorylated tau levels and the p-tau/amyloid-beta_1-42_ ratio (Tsolaki et al., 2014; Rossini et al., 2020c). Recent MEG studies, by mapping regional neurophysiological synchrony patterns and directly comparing these spatial maps to PET tracer uptake for tau (e.g., flortaucipir tracers) and amyloid-beta, revealed that alpha hyposynchrony colocalizes with tau protein deposits while delta-theta hypersynchrony colocalizes with both tau and amyloid-beta deposits, with alpha hyposynchrony showing strong correlation with cognitive decline severity (Ranasinghe et al., 2020).

Numerous studies have reported correlations between EEG changes and neuroimaging findings. In aMCI and AD subjects, cortical gray matter volume is positively correlated with diffuse alpha activity and negatively correlated with diffuse delta activity. Beyond cortical gray matter, global alpha and delta power correlate with thalamic and basal ganglia gray matter volumes, subcortical white matter integrity, and hippocampal atrophy levels. Combining MRI, PET, P300 analysis, and EEG spectral power analysis has enhanced diagnostic accuracy for dementia. Relative theta power has been shown to predict dementia severity with accuracy comparable to PET markers (Ranasinghe et al., 2020; Rossini et al., 2020a).

*APOE* genotype influences EEG characteristics in AD (Jelic et al., 1996; Lehtovirta et al., 2000). Carriers of the ε4 allele demonstrate more pronounced slow-wave activity, which appears to correlate with the degree of cholinergic deficit (Babiloni et al., 2004). Alpha1 activity in parietooccipital cortex is more reduced in ε4 carriers compared to non-carriers (Hatz et al., 2013a). Phase synchronization in the alpha2 band decreases in lateral frontal and parietotemporal regions in ε4 carriers. Combining qEEG analysis with *APOE* genotyping may enhance early-stage AD identification and differentiation from amnestic MCI (Ponomareva et al., 2003; Hatz et al., 2013b).

### 4.2 EEG characteristics in dementia with Lewy bodies

DLB produces the most distinctive EEG pattern among the neurodegenerative dementias, with high diagnostic accuracy for differentiating DLB from AD (Walker et al., 2000; Bonanni et al., 2008). The hallmark finding is slowing of the dominant background rhythm to below 8 Hz, present in approximately 88% of DLB patients (Bonanni et al., 2008). Mean dominant frequency in DLB (7.4 ± 1.6 Hz) was significantly lower than in AD (8.3 ± 0.6 Hz), with minimal overlap between groups. Mean peak frequency in DLB ranges from 6.7 to 7.5 Hz compared to 7.5–8.8 Hz in AD (Andersson et al., 2008). This finding has been incorporated into the fourth consensus criteria for DLB as a supportive diagnostic biomarker (McKeith et al., 2017).

Beyond dominant rhythm slowing, DLB demonstrates several additional characteristic features (Briel et al., 1999; Kai et al., 2005). Frontal intermittent rhythmic delta activity (FIRDA) appears in 22% of patients with MCI due to Lewy body disease but is absent in MCI due to AD (van der Zande et al., 2020). Dominant frequency variability, quantified as fluctuation in the frequency of the posterior dominant rhythm across the recording, correlates with cognitive fluctuations that represent a core diagnostic feature of DLB (Walker et al., 2000). This variability may reflect the moment-to-moment instability of thalamocortical circuits in DLB (Andersson et al., 2008).

Quantitative analyses achieve high diagnostic accuracy for discriminating DLB from AD (Bonanni et al., 2015; Stylianou et al., 2018). Automated classification algorithms using spectral features have demonstrated sensitivity and specificity approaching 90% (Bonanni et al., 2015). This performance exceeds that of many other DLB biomarkers and approaches the accuracy of dopamine transporter imaging for identifying Lewy body pathology (McKeith et al., 2017). EEG thus represents a relatively inexpensive and widely available biomarker with substantial diagnostic utility in the workup of dementia with fluctuations or visual hallucinations.

The presence of REM sleep without atonia, detected through polysomnography or overnight video-EEG, provides additional diagnostic value (Iranzo et al., 2006; Boeve et al., 2007). REM sleep behavior disorder precedes DLB onset by years to decades in most cases and demonstrates high specificity for synucleinopathies (Postuma et al., 2019). Sleep architecture abnormalities in DLB include reduced REM sleep percentage, increased REM latency, and disrupted sleep spindle generation, all reflecting dysfunction of brainstem and thalamic circuits affected early in Lewy body disease (Pao et al., 2013).

### 4.3 EEG characteristics in frontotemporal dementia

In contrast to AD and DLB, FTD demonstrates relatively preserved background EEG activity, particularly in early disease stages (Lindau et al., 2003; Chan et al., 2004). Visual EEG inspection may appear normal or near normal in behavioral variant FTD, even when significant frontal and anterior temporal atrophy is evident on neuroimaging. This preservation of posterior rhythms reflects the relative sparing of parietooccipital cortex and thalamocortical networks in typical FTD (Jóhannesson et al., 1977; Yu et al., 2016; Olğun et al., 2024).

Quantitative analysis reveals more subtle abnormalities (Nishida et al., 2011; Caso et al., 2012; Cecchetti et al., 2024; Benussi et al., 2026). Decreased alpha band power is observed in orbitofrontal and anterior temporal regions, corresponding to areas of maximal pathology. Compared to AD, FTD shows less posterior delta activity and relative preservation of synchronization in fast frequencies (Lindau et al., 2003). Frontal-temporal hypoconnectivity patterns, reflecting disruption of frontostriatal and frontolimbic circuits, emerge as specific biomarkers for behavioral variant FTD (Yu et al., 2016; Dottori et al., 2017).

The F-theta/T-alpha ratio, comparing frontal theta power to temporal alpha power, shows promise as a quantitative marker differentiating FTD from AD (Chang and Chang, 2023; Wang et al., 2024). This ratio captures the relatively greater frontal dysfunction in FTD while reflecting the temporoparietal predominance of AD pathology. However, overlap exists between conditions, and EEG alone cannot reliably distinguish FTD from AD at the individual patient level without integration with clinical, neuropsychological, and neuroimaging data (Caso et al., 2012).

### 4.4 Epileptiform abnormalities and detection methods

Interictal epileptiform discharges (IEDs) in AD demonstrate predominantly left temporal localization (57%), with bitemporal involvement in 26% and other distributions comprising the remainder (Vossel et al., 2016a). Temporal intermittent rhythmic delta activity (TIRDA) serves as a marker of temporal lobe hyperexcitability and should raise consideration of underlying epileptiform susceptibility even in the absence of definitive spikes or sharp waves (Reiher et al., 1989).

Detection rates for epileptiform abnormalities vary dramatically by recording duration and modality (Vossel et al., 2016a; Lam et al., 2020). Routine 20–30-min EEG detects IEDs in only 2-3% of AD patients, reflecting both the low baseline probability of capturing infrequent discharges and the sleep-state preference of most epileptiform activity (Liedorp et al., 2010). 24-h ambulatory EEG increases yield to 22-54% by capturing extended wake–sleep cycles (Brunetti et al., 2020; Lam et al., 2020). MEG provides complementary sensitivity, detecting 21% of IEDs not visible on simultaneous scalp EEG, likely reflecting superior detection of deep sources such as hippocampal and mesial temporal activity (Vossel et al., 2016a).

The main electroencephalographic elements considered to have epileptiform significance include spikes (duration 20–70 ms), sharp waves (duration 70–200 ms), polyspike complexes, spike-and-wave complexes, and sharp wave-slow wave complexes (Noachtar and Rémi, 2009; Kane et al., 2017). These elements are defined by their clear distinction from background activity, pointed peak morphology, and typically negative polarity. Correct identification requires expertise in distinguishing true epileptiform discharges from artifacts and normal variants, which can be challenging in elderly patients with various sources of EEG artifact (Tatum, 2021).

Sleep activation is critical for epileptiform detection in dementia (Horváth et al., 2018; Brunetti et al., 2020). The majority of IEDs in AD occur during non-REM sleep, particularly slow-wave sleep, and may be entirely absent during wakefulness. This sleep-state predilection has implications for monitoring strategies: extended ambulatory recordings with adequate sleep capture provide substantially higher yield than repeated daytime routine EEGs (Lam et al., 2020). Overnight video-EEG monitoring represents the gold standard for comprehensive evaluation when epilepsy is suspected (McBride et al., 2002).

## 5 Pathophysiological mechanisms of epileptogenesis

The mechanisms underlying epileptogenesis in neurodegenerative dementias involve complex interactions between pathological protein aggregates, neurotransmitter systems, and neural network function (Palop and Mucke, 2016; Vossel et al., 2017a). Converging evidence from animal models, human biomarker studies, and cortical excitability assessments points to a common theme: disruption of the balance between neuronal excitation and inhibition, leading to network hyperexcitability and hypersynchrony that manifests as epileptiform activity and clinical seizures (Busche and Konnerth, 2016; Zott et al., 2019).

### 5.1 Role of amyloid-beta

Amyloid-beta oligomers represent the species most directly implicated in neuronal hyperexcitability (Busche and Konnerth, 2016; Zott et al., 2019). Multiple mechanisms have been identified through which Abeta promotes seizure susceptibility. First, amyloid-beta selectively upregulates Nav1.6 voltage-gated sodium channels, causing membrane depolarization and lowering the threshold for action potential generation (Wang et al., 2016; Ciccone et al., 2019). Second, amyloid-beta disrupts presynaptic GABA release from fast-spiking parvalbumin-positive interneurons, weakening inhibitory control over pyramidal cell activity (Verret et al., 2012; Palop and Mucke, 2016; Smeralda et al., 2024). Third, amyloid-beta impairs astrocytic glutamate reuptake, leading to elevated extracellular glutamate and excitotoxicity (Li et al., 2009; Zott et al., 2019).

Two-photon calcium imaging studies in AD mouse models have provided compelling evidence for early network hyperexcitability (Busche et al., 2008; Busche and Konnerth, 2016). In APP23xPS45 mice, hyperactive neurons appear in hippocampal CA1 as early as 1.5 months of age, before plaque formation and overt memory impairment (Busche et al., 2008). This hyperactivity is reversible with acute gamma-secretase inhibition, confirming that soluble amyloid-beta species rather than deposited plaques drive the phenomenon. The temporal sequence supports amyloid-beta-induced hyperexcitability as an early event in AD pathogenesis rather than a late consequence of neuronal loss (Busche and Konnerth, 2016).

Network hypersynchrony emerges as a consequence of hyperactive individual neurons (Palop et al., 2007; Verret et al., 2012). In Tg2576 mice, spontaneous interictal spikes, increased seizure susceptibility, and ectopic neuropeptide Y expression in mossy fibers appear by 1.5 months, before memory impairments manifest at 3 months (Palop et al., 2007). This hypersynchrony affects the normal physiological rhythms that support cognition, disrupting sharp wave-ripple activity essential for memory consolidation and replacing it with pathological epileptiform discharges (Buzsáki, 2015).

The particular relevance of amyloid-beta for epileptogenesis is supported by the lower seizure frequency in non-amyloid dementias (Beagle et al., 2017). Tauopathies such as progressive supranuclear palsy and corticobasal degeneration, as well as synucleinopathies such as Parkinson’s disease, demonstrate substantially lower seizure risk than AD despite comparable or greater neuronal loss in some brain regions (Beagle et al., 2017). This pattern suggests that amyloid-beta-specific mechanisms, rather than neurodegeneration *per se*, drive the elevated epilepsy risk in AD (Sanchez et al., 2012).

### 5.2 Role of tau protein

While Abeta oligomers may initiate hyperexcitability, tau protein plays a permissive and potentially independent role in seizure generation (Roberson et al., 2007; Holth et al., 2013). Studies in tau knockout mice have provided striking evidence: hAPP-J20 mice lacking tau demonstrate normalized inhibitory/excitatory balance, rescue from early lethality, and improved cognition compared to hAPP-J20 mice with normal tau expression (Roberson et al., 2007). Antisense oligonucleotide reduction of tau prevents chemically induced seizures in wild-type rodents and spontaneous seizures in SCN1A mutant mice (genetic model of Dravet syndrome), suggesting tau-directed therapies could have broad antiepileptic potential (DeVos et al., 2013; Gheyara et al., 2014; Putra et al., 2020).

Hyperphosphorylated tau promotes excitotoxic damage and increases GABA-A receptor-mediated hyperexcitability even in the absence of pathological amyloid-beta (Tai et al., 2016; Sánchez et al., 2018). This observation has implications for understanding seizures in tauopathies that lack amyloid pathology. Conversely, epileptic activity itself promotes tau hyperphosphorylation through activation of kinases including GSK-3beta and CDK5, creating a pathological feedforward cycle whereby seizures beget more tau pathology, which begets more seizures (Liang et al., 2009; Tai et al., 2016).

Evidence from human surgical specimens in temporal lobe epilepsy has demonstrated variable degrees of tau accumulation in resected tissue, but seems to support this bidirectional relationship (Thom et al., 2011; Tai et al., 2016; Prada Jardim et al., 2018; DePaula-Silva et al., 2021; Toscano et al., 2023). Autopsy studies of temporal lobectomies performed for pharmacoresistant epilepsy reveal epilepsy-related tauopathy in temporal lobe tissue, with the extent of tau pathology correlating with postoperative verbal memory decline (Tai et al., 2016). CSF total tau (but not phosphorylated tau or Abeta42) associates with seizure probability in AD patients, providing a potential biomarker for identifying those at highest epilepsy risk (Vossel et al., 2013a).

### 5.3 Role of alpha-synuclein

Alpha-synuclein pathology in DLB and Parkinson’s disease contributes to EEG abnormalities through mechanisms partially distinct from amyloid-beta and tau (Morris et al., 2015; Franciotti et al., 2020; Peters et al., 2020). Transgenic mouse models overexpressing human alpha-synuclein demonstrate EEG slowing matching patterns observed in human DLB, along with frank seizures and depletion of calbindin in the dentate gyrus (Morris et al., 2015). Calbindin loss is a recognized marker of chronic seizures and reflects calcium-mediated excitotoxic stress (Sloviter et al., 1991).

The A53T alpha-synuclein mutation, associated with familial Parkinson’s disease, causes long-term potentiation deficits coinciding with EEG abnormalities in mouse models (Teravskis et al., 2018; Peters et al., 2020). Preformed alpha-synuclein fibrils sequester endogenous alpha-synuclein from presynaptic terminals, reducing excitatory tone and compromising functional connectivity (Volpicelli-Daley et al., 2011). This mechanism may contribute to the cognitive fluctuations, characteristic of DLB, wherein moment-to-moment variation in synaptic alpha-synuclein availability produces corresponding fluctuations in cortical arousal and attention (Peraza et al., 2014).

### 5.4 Role of TDP-43

TDP-43 proteinopathy, the predominant pathology in most FTD cases and a frequent co-pathology in AD, has been linked to seizure susceptibility (Nelson et al., 2019; Rocca et al., 2025). Brain bank studies demonstrate higher TDP-43 pathology scores in middle temporal gyrus of AD donors with coexisting epilepsy compared to those without (Nelson et al., 2019; Rocca et al., 2025). Mice expressing human TDP-43 with nuclear localization signal mutations develop hyperexcitability and generalized seizures, providing experimental support for TDP-43’s epileptogenic potential (Dyer et al., 2021; Ra et al., 2024; Joseph et al., 2025; Rodemer et al., 2025).

The relatively lower seizure risk in FTD compared to AD, despite frequent TDP-43 pathology, may reflect the regional distribution of pathology (frontal predominant in FTD versus temporal predominant in AD) or differential interactions with other pathological proteins (Mackenzie and Neumann, 2016). The frontal lobes are less intrinsically epileptogenic than temporal structures, and the absence of amyloid pathology in most FTD cases may limit seizure generation despite TDP-43 dysfunction (Engel, 2001).

### 5.5 Transcranial magnetic stimulation evidence for cortical excitability changes

Transcranial magnetic stimulation (TMS) provides direct *in vivo* assessment of cortical excitability in dementia patients, revealing distinct profiles across neurodegenerative conditions (Di Lazzaro et al., 2021; Oberman and Benussi, 2023; Vucic et al., 2023; see Table 3). Meta-analysis of 61 TMS studies demonstrates that AD exhibits increased motor cortex excitability, manifested as lower resting motor threshold, along with reduced short interval intracortical inhibition (SICI) reflecting GABAergic dysfunction (Chou et al., 2022). Short-latency afferent inhibition (SAI), a TMS paradigm reflecting cholinergic function, is markedly reduced in both AD and DLB, consistent with the cholinergic deficits central to both conditions (Di Lazzaro et al., 2004, 2006; Nardone et al., 2006; Benussi et al., 2015, 2017a, 2018a,b, 2020d, 2022b,[Bibr ref35][Bibr ref176], 2019). However, intracortical facilitation (ICF), reflecting glutamatergic function, shows divergent patterns: AD typically demonstrates preserved or enhanced ICF, whereas DLB shows reduced ICF (Di Lazzaro et al., 2007; Benussi et al., 2018b, 2022c; Rizzardi et al., 2025). This difference may relate to differential involvement of glutamatergic systems, through NMDA receptor–mediated glutamatergic transmission, or reflect the impact of alpha-synuclein on presynaptic glutamate release in DLB. In fact, in AD Aβ-induced oxidative stress impairs astrocytic glutamine synthetase, disrupting the glutamate-glutamine cycle and promoting extracellular glutamate accumulation with enhanced NMDA-mediated transmission (Aksenov et al., 1995). This relative hyperglutamatergic state may account for preserved or increased ICF. In contrast, in DLB, aggregated and phosphorylated *α*-synuclein interferes with presynaptic vesicle dynamics and glutamate release, leading to reduced excitatory drive (Grosso et al., 2014; Ferrari et al., 2023; Guan et al., 2025). This presynaptic hypoglutamatergic dysfunction may underlie the decreased ICF observed in DLB.

**Table 3:** TMS cortical excitability profiles across neurodegenerative dementias.

TMS parameter	Interpretation	Alzheimer’s disease	DLB	FTD
Motor threshold	Cortical excitability	Decreased (increased excitability)	Normal	Normal
SICI	GABA-A function	Decreased	Decreased	Decreased
ICF	Glutamatergic function	Variable	Decreased	Decreased
SAI	Cholinergic function	Markedly decreased	Markedly decreased	Preserved

TMS, transcranial magnetic stimulation; DLB, dementia with Lewy bodies; FTD, frontotemporal dementia; SICI, short-interval intracortical inhibition; GABA-A, gamma-aminobutyric acid type A; ICF, intracortical facilitation; SAI, short-latency afferent inhibition.

FTD presents a distinct TMS profile with relatively preserved SAI (consistent with intact cholinergic pathways) but reduced glutamatergic and GABAergic function (Burrell et al., 2011; Benussi et al., 2016, 2017b, 2018b, 2019a,b,c, 2020a,b,c,e,f, 2021; Gazzina et al., 2018; Bracca et al., 2023). This pattern provides differential diagnostic value and suggests that the mechanisms of cortical dysfunction differ between FTD and AD/DLB despite superficially similar cognitive impairments (Borroni et al., 2018).

### 5.6 Network-level mechanisms

Beyond cellular and molecular mechanisms, network-level dysfunction provides the substrate for epileptiform activity in dementia (Noebels, 2011; Palop and Mucke, 2016). The hippocampus, one of the earliest and most severely affected structures in AD, is also one of the most intrinsically epileptogenic regions of the brain (Ramey et al., 2013; Núñez-Ochoa et al., 2021). Seizures occurring early in AD likely originate from the mesial temporal lobe, where transient epileptic discharges may impair cognitive abilities and, through persistent damage to hippocampal circuits, lead to progressive memory loss (Noebels, 2011).

Aberrant neurogenesis in the dentate gyrus contributes to network reorganization in AD (Palop et al., 2007; Li et al., 2008). Newly generated neurons demonstrate reduced probability of maturing into GABAergic interneurons, further weakening inhibitory control. These cells also show abnormal integration into existing circuits, potentially forming aberrant excitatory connections that lower seizure threshold. Similar patterns of aberrant neurogenesis have been described in chronic temporal lobe epilepsy, suggesting convergent mechanisms (Parent et al., 1997).

Failure of the dentate gyrus gate, a physiological mechanism that normally filters cortical inputs to prevent their transmission to hippocampal CA3 and CA1, may permit pathological synchronization (Heinemann et al., 1992). In healthy function, the dentate gyrus sparse coding pattern prevents pattern completion in CA3 from being triggered by noisy or incomplete inputs. AD pathology disrupts this gating function, potentially allowing subcortical or neocortical activity to drive hippocampal synchronization and seizure generation (Palop et al., 2007).

## 6 The system epilepsy framework

**Figure 1:**
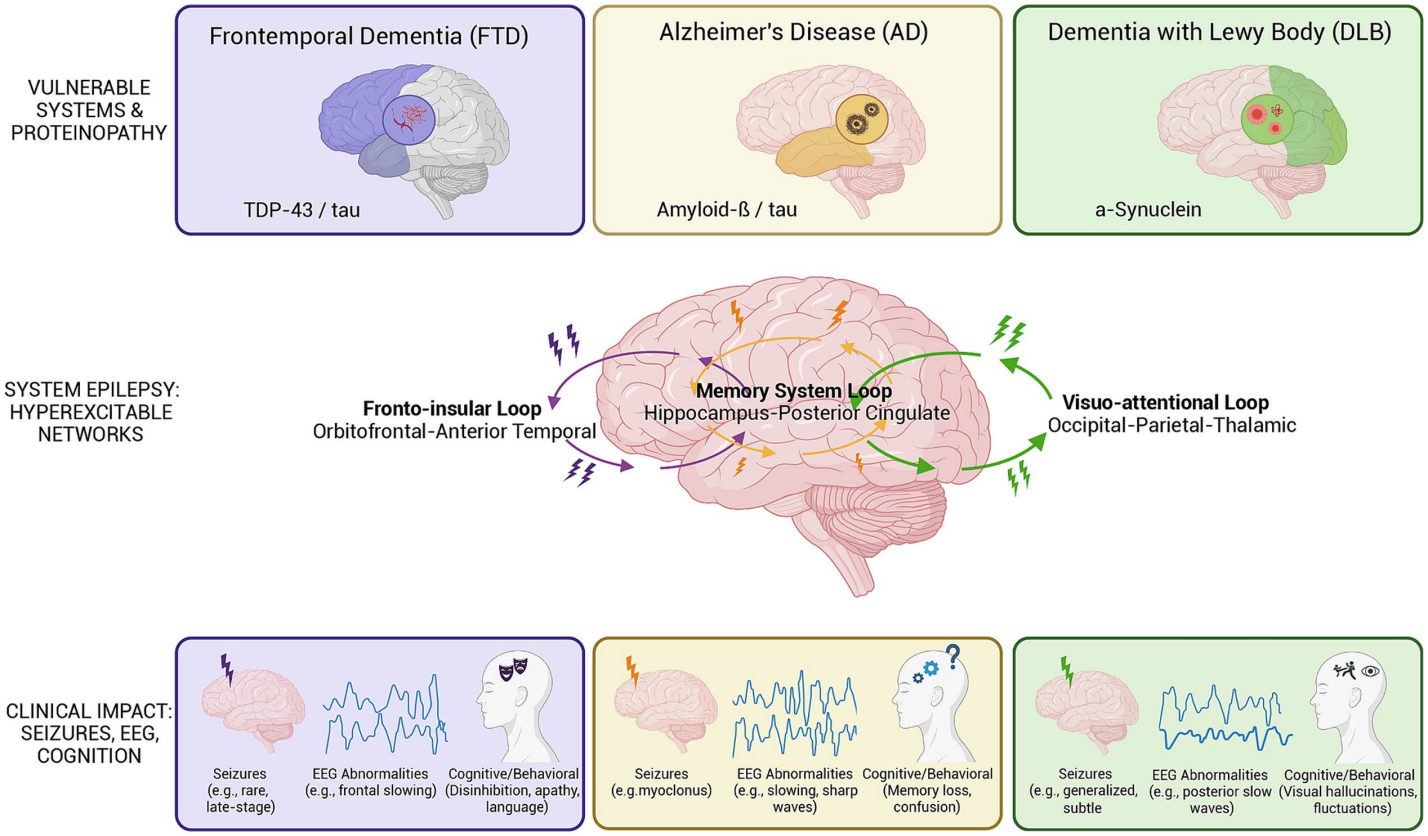
Disease-specific network vulnerability and clinical manifestations of epilepsy in neurodegenerative dementias. Schematic overview illustrating the system epilepsy framework across the three major neurodegenerative dementias. Top row: Vulnerable brain systems and underlying proteinopathies. Middle row: Disease-specific functional loops implicated in network hyperexcitability. Bottom row: Clinical manifestations including seizure phenotypes, characteristic EEG abnormalities, and cognitive/behavioral features for each condition. AD, Alzheimer’s disease; DLB, dementia with Lewy bodies; EEG, electroencephalography; FTD, frontotemporal dementia; TDP-43, TAR DNA-binding protein 43.

The concept of system epilepsies, articulated by Avanzini and colleagues in their influential 2012 Epilepsia paper, describes epilepsies arising from an enduring propensity to generate seizures involving functionally characterized brain systems rather than discrete focal lesions (Avanzini et al., 2012). This framework, originally developed to explain self-limited childhood epilepsies, offers a compelling lens through which to understand epilepsy associated with neurodegenerative dementias (see Figure 1).

### 6.1 Characteristics of classical system epilepsies

Childhood system epilepsies including self-limited epilepsy with centrotemporal spikes (Rolandic epilepsy), Panayiotopoulos syndrome, and childhood absence epilepsy share several defining characteristics (Capovilla et al., 2009; Manganotti and Del Felice, 2013; Koutroumanidis et al., 2017). First, they demonstrate age-dependent expression, appearing within specific developmental windows and typically remitting by adulthood (Specchio et al., 2022). Second, they involve functional neural networks (sensorimotor, autonomic, thalamocortical absence circuits) rather than structural lesions identifiable on neuroimaging (Panayiotopoulos et al., 2008; Lemke et al., 2012). Third, they exhibit genetic predisposition, with heritability reflecting polygenic susceptibility to network hyperexcitability (Vadlamudi et al., 2006; Rudolf et al., 2009; Thakran et al., 2020). Fourth, they typically carry favorable prognosis with high rates of seizure remission (Panayiotopoulos et al., 2008; Lamberink et al., 2017). Fifth, the brain appears structurally normal on routine neuroimaging (Panayiotopoulos et al., 2008; Jacobs, 2020). Sixth, seizure semiology reflects the functional specialization of involved networks (Manganotti and Zanette, 2000; Manganotti et al., 2001, 2004; Brigo et al., 2012, 2013; Jacobs, 2020).

In Rolandic epilepsy, TMS studies demonstrate increased motor intracortical excitability, suggesting that the perisylvian somatosensory-motor network exists in a state of enhanced excitability that predisposes to centrotemporal spikes and sensorimotor seizures (Nezu et al., 1997; Manganotti and Zanette, 2000; Baumer et al., 2020). This excitability normalizes with age, correlating with clinical remission. The epilepsy thus represents dysfunction of a maturating functional system rather than a fixed structural lesion (Panayiotopoulos et al., 2008).

### 6.2 Application to dementia-associated epilepsy

Multiple lines of evidence support conceptualizing dementia-associated epilepsy within this framework rather than as classical focal symptomatic epilepsy arising from discrete lesions. Importantly, the system epilepsy framework does not rely simply on the absence of a structural lesion or on the recognition that seizures arise from distributed networks, concepts already embedded in modern epilepsy classification. Rather, in dementia, epileptogenicity may be intrinsic to the progressive dysfunction of a physiologically defined neural system. The memory-limbic network in AD or the distributed cortico-subcortical systems affected in DLB may become inherently unstable due to selective molecular and synaptic vulnerability. Seizures thus emerge as a property of system-level degeneration rather than a secondary consequence of focal injury. This perspective reframes seizures as part of the disease phenotype, supporting systematic surveillance and earlier network-targeted therapeutic strategies.

First, excitability changes in dementia-associated epilepsy are characteristically bilateral rather than lateralized. TMS studies demonstrate motor cortex hyperexcitability in AD with reduced short interval intracortical inhibition (SICI) affecting both hemispheres, and EEG abnormalities typically shift between hemispheres and regions rather than remaining fixed to a single focus (Vucic et al., 2023; Benussi and Vucic, 2026). This bilateral, fluctuating pattern contrasts sharply with truly focal epilepsies, where excitability changes are lateralized to the epileptogenic zone.

Second, the pathophysiology of dementia-associated epilepsy operates at the network level. Seizures in dementia arise from dysfunction within large-scale networks, including the default mode network and limbic memory systems, rather than from discrete structural damage. Widespread subclinical epileptiform activity correlates with diffuse cognitive decline across multiple domains, not merely with impairment of functions localized to a putative epileptogenic focus (Vossel et al., 2016b; Ranasinghe et al., 2022).

Third, no circumscribed epileptogenic lesion is typically identifiable. AD involves diffuse pathology affecting multiple interconnected regions, and while mesial temporal structures may be preferentially involved early in the disease course, the distribution of epileptiform activity often exceeds what would be expected from a single temporal focus (Samudra et al., 2023; Zawar and Kapur, 2023).

Fourth, dementia-associated epilepsy exhibits age-dependent expression that parallels, in reverse, the childhood-specific expression of pediatric system epilepsies. Rather than reflecting maturation of inhibitory systems, the late-life emergence of seizures reflects degeneration of networks that previously maintained stable excitability (Vossel et al., 2013b; Johnson et al., 2020). This represents dysfunction of degenerating memory and cognitive networks, analogous to the dysfunction of maturing sensorimotor networks in Rolandic epilepsy.

**Figure 2:**
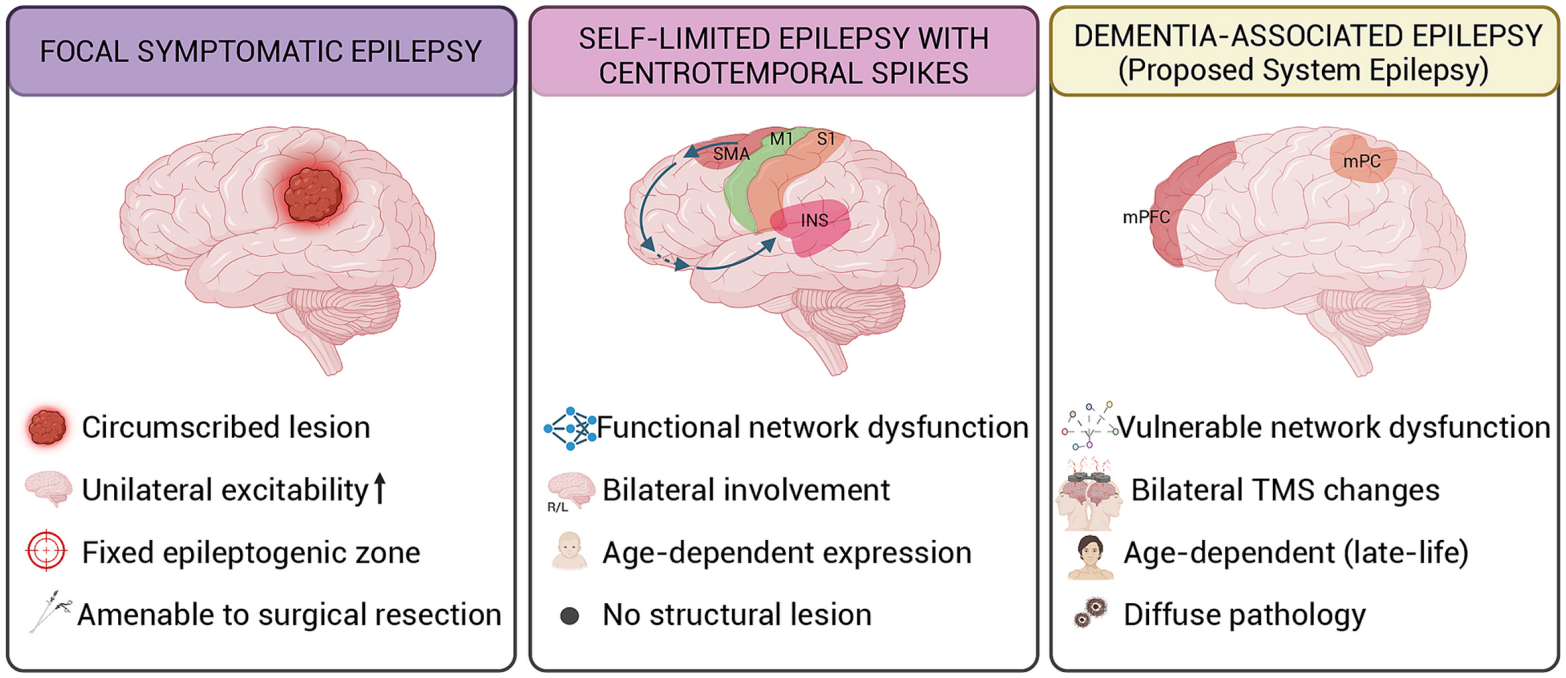
Conceptual comparison of three epilepsy paradigms. INS, insula; M1, primary motor cortex; mPC, medial parietal cortex; mPFC, medial prefrontal cortex; R/L, right/left; S1, primary somatosensory cortex; SMA, supplementary motor area; TMS, transcranial magnetic stimulation.

Finally, the brain systems involved in AD-associated epilepsy, memory networks, limbic circuits, and cholinergic systems, represent functionally characterized systems consistent with the system epilepsy concept. Seizures and epileptiform activity preferentially emerge from and disrupt the functions of these vulnerable networks (Vossel et al., 2017b; Toniolo et al., 2020; see Figure 2).

### 6.3 Implications of the system epilepsy paradigm

Reconceptualizing dementia-associated epilepsy as a system epilepsy has several important implications. First, it shifts clinical approach away from a purely focal lesion model traditionally used in some epilepsies toward understanding network hyperexcitability as an intrinsic and potentially modifiable feature of neurodegeneration (Vossel et al., 2017a). In this context, the goal is not to identify resectable epileptogenic zones, which would rarely be appropriate in patients with progressive neurodegenerative disease, but to recognize diffuse network dysfunction as a core pathophysiological process. Surgical approaches that succeed in focal epilepsies are unlikely to benefit patients with diffuse network dysfunction.

Second, this framework supports lower thresholds for extended EEG monitoring given the subclinical nature of much epileptiform activity (Lam et al., 2020). If network hyperexcitability represents an intrinsic feature of dementia pathophysiology rather than a rare complication, then systematic screening may identify a substantial proportion of patients who could benefit from intervention.

Third, the system epilepsy framework provides rationale for network-targeted therapeutic approaches and earlier intervention (Bakker et al., 2012). If hyperexcitability contributes to disease progression through mechanisms such as enhanced amyloid release, tau spreading, and excitotoxic injury, then treatment aimed at normalizing network function could potentially modify disease course rather than merely controlling a symptomatic complication (Sanchez et al., 2012).

## 7 Therapeutic implications

Management of epilepsy in dementia patients requires careful consideration of drug selection, potential cognitive effects, drug–drug interactions, and the possibility that antiseizure treatment may provide benefits beyond seizure control (Vossel et al., 2017a; Sen et al., 2020). The elderly population with dementia presents unique pharmacological challenges, including altered pharmacokinetics, polypharmacy, and heightened sensitivity to cognitive side effects (Leppik, 2006; Brodie et al., 2009).

### 7.1 Antiseizure medication selection

Second-generation antiseizure medications (ASMs) are preferred in elderly patients with dementia due to favorable pharmacokinetic profiles and minimal cognitive effects (Brodie et al., 2009; Cretin, 2018). The International League Against Epilepsy (ILAE) Task Force on Epilepsy in the Elderly concluded that levetiracetam has the strongest evidence for long-term seizure freedom and favorable tolerability in older adults and is therefore a preferred first-line option (Piccenna et al., 2023).

Levetiracetam offers several advantages in the dementia population: no hepatic metabolism eliminating drug–drug interactions with cholinesterase inhibitors and memantine; renal elimination with straightforward dose adjustment for common age-related renal impairment; no enzyme induction or inhibition; favorable cognitive profile with some evidence of cognitive benefit in certain populations; and particular efficacy in patients over 65 years of age (Belcastro et al., 2007; Cumbo and Ligori, 2010). Starting doses should be conservative (125–250 mg twice daily) with gradual titration.

Lamotrigine represents an alternative first-line option with ILAE/American Academy of Neurology-recommended efficacy for focal seizures (Kanner et al., 2018). It demonstrates minimal cognitive deficits and offers potential mood stabilization benefits relevant to the high prevalence of depression in dementia (Brodie et al., 1999). However, slow titration is required to minimize Stevens-Johnson syndrome risk, making lamotrigine less suitable when rapid seizure control is needed.

Brivaracetam, with approximately 10-fold higher SV2A affinity than levetiracetam, provides an excellent option when levetiracetam behavioral side effects emerge (Klein et al., 2015). Irritability affects only 5.6% with brivaracetam compared to 9.9% with levetiracetam. Conversion can be accomplished at ratios of 10:1 to 15:1 (levetiracetam to brivaracetam; Steinhoff et al., 2021).

Medications to avoid in dementia patients include older enzyme-inducing ASMs (phenytoin, phenobarbital, carbamazepine) due to drug interactions and cognitive effects; topiramate, which has clear evidence of cognitive impairment; and benzodiazepines, which risk paradoxical reactions, cognitive impairment, falls, and respiratory depression (Loring et al., 2007; Perucca and Gilliam, 2012). Valproic acid, while effective, carries risks of tremor exacerbation, Parkinsonism, and potential acceleration of brain atrophy that limit its use in neurodegenerative conditions (Armon et al., 1996; Sills and Rogawski, 2020).

### 7.2 Levetiracetam: beyond seizure control

Levetiracetam has emerged as a molecule of particular interest in dementia not only for seizure control but for potential disease-modifying effects (Sanchez et al., 2012; Vossel et al., 2021). The drug exerts its antiseizure action through binding to synaptic vesicle protein 2A (SV2A), which modulates neurotransmitter release (Lynch et al., 2004). Preclinical evidence demonstrates multiple potentially beneficial mechanisms: reduction of amyloid-beta_1-42_ levels through decreased amyloidogenic APP processing; suppression of neuronal network dysfunction in AD mouse models including hAPP-J20 and APP/PS1 lines; reversal of synaptic and cognitive deficits; normalization of microglial phenotypes; and improvement of mitochondrial dysfunction (Sanchez et al., 2012; Shi et al., 2013; Zheng et al., 2022).

The LEV-AD trial (Vossel et al., 2021), a phase 2a double-blind placebo-controlled crossover study in 34 adults with AD, evaluated the cognitive effects of levetiracetam. While the primary endpoint (NIH-EXAMINER composite) was not met in the overall population, prespecified analysis revealed that levetiracetam improved executive function and spatial memory specifically in AD patients with epileptiform activity, comprising 38% of the cohort. This finding suggests that treatment effects may be most pronounced in the subgroup with demonstrable network hyperexcitability.

Another study demonstrated improved attention and verbal fluency in AD patients with seizures treated with levetiracetam over 1 year of follow-up (Cumbo and Ligori, 2010). These cognitive benefits may reflect reduction of subclinical epileptiform activity that disrupts attention and memory consolidation or alternatively may represent direct neuroprotective effects of SV2A modulation independent of epileptiform activity suppression (Koh et al., 2010).

Behavioral side effects of levetiracetam require monitoring, particularly irritability (9.9%) and aggression (2.6%; Mula et al., 2004). These effects typically emerge within the first weeks of treatment and may resolve with dose reduction or time. In patients who cannot tolerate levetiracetam due to behavioral side effects, brivaracetam offers an alternative SV2A ligand with lower behavioral adverse event rates (Klein et al., 2015).

### 7.3 Clinical trials targeting hyperexcitability

The HOPE4MCI trial tested AGB101, a low-dose extended-release formulation of levetiracetam (220 mg daily), in 164 amyloid-positive MCI patients over 78 weeks (Mohs et al., 2024). The primary endpoint of change in Clinical Dementia Rating Sum of Boxes (CDR-SB) was not met (1.12 vs. 1.22 for placebo, non-significant), though AGB101 showed 8% less worsening. Notably, subgroup analysis revealed that *APOE4* non-carriers experienced 40% less decline in CDR-SB, suggesting potential differential response by genotype warranting further investigation.

Foundational work by Bakker et al. (2012) demonstrated that low dose levetiracetam normalized hippocampal hyperactivity on functional MRI and improved pattern separation performance in amnestic MCI patients. This proof-of-concept study established that pharmacological modulation of hippocampal hyperexcitability is feasible and can produce measurable cognitive benefits.

Ongoing phase 2 trials include a Walter Reed study (NCT04004702) assessing levetiracetam for neuropsychiatric symptoms in AD patients with epileptiform activity, and a Beth Israel Deaconess trial (NCT03875638) evaluating low-dose levetiracetam as a disease-modifying agent. Additionally, a phase 2 AGB101 trial initiated in 2023 with entorhinal cortex volume change as the primary endpoint (completion expected 2028; NCT05986721).

### 7.4 Treatment of subclinical epileptiform activity

Whether to treat subclinical epileptiform activity in the absence of clinical seizures remains an area of active investigation (Lam et al., 2020; Vossel et al., 2021). The strong correlation between spike frequency and cognitive decline rate demonstrated by Horvath et al. (2021) suggests that suppression of epileptiform activity could meaningfully slow progression. However, definitive evidence from randomized controlled trials is not yet available.

Several expert groups have proposed considering empirical treatment when subclinical epileptiform activity is associated with cognitive fluctuations, unexplained confusion episodes, or accelerated cognitive decline, though formal guidelines have not yet been issued (Vossel et al., 2017a; Sen et al., 2020). Low dose levetiracetam (125 mg twice daily) represents a reasonable starting point given its favorable side effect profile. Response should be assessed at 4-12 weeks through clinical assessment and, ideally, repeat EEG monitoring to confirm reduction in epileptiform activity.

For patients with documented subclinical epileptiform activity but stable cognition, the decision is more individualized (Lam et al., 2020). The potential benefit of preventing epileptiform activity-mediated cognitive decline must be weighed against medication burden, cost, and potential side effects. Shared decision-making with patients and caregivers, informed by the emerging evidence linking epileptiform activity to accelerated decline, is appropriate.

### 7.5 Clinical monitoring recommendations

Given the high prevalence of subclinical epileptiform activity and its prognostic significance, we recommend expanded use of prolonged EEG monitoring in dementia patients (Vossel et al., 2016a; Lam et al., 2020). Twenty-four-hour ambulatory EEG should be considered in patients with AD presenting with unexplained cognitive fluctuations, episodic confusion, or suspected seizure-like events. This monitoring should include adequate sleep capture given the sleep-state predilection of most epileptiform activity (Horváth et al., 2018).

Routine EEG remains valuable for its diagnostic contribution to dementia differential diagnosis, particularly for identifying the characteristic slowing of DLB (Bonanni et al., 2008), but its sensitivity for epileptiform activity detection is limited (Liedorp et al., 2010). When routine EEG is negative but clinical suspicion for epilepsy remains high, extended monitoring should be pursued rather than excluding an epileptic contribution.

Repeat monitoring may be indicated in patients with progressive cognitive decline or emergence of new symptoms suggestive of seizure activity (Vossel et al., 2017a). The intermittent nature of epileptiform discharges means that a single negative study does not exclude their presence, particularly if recording duration was limited or sleep capture was inadequate (Lam et al., 2017).

## 8 Conclusion and future directions

Epilepsy and epileptiform activity represent underrecognized yet clinically significant features of neurodegenerative dementias with substantial impact on disease trajectory (Vossel et al., 2017a; Horvath et al., 2021). The convergence of evidence from epidemiological studies, extended neurophysiological monitoring, TMS cortical excitability assessments, and mechanistic investigations supports a fundamental reconceptualization of dementia-associated epilepsy as a system-level phenomenon arising from dysfunctional neural networks (Avanzini et al., 2012; Palop and Mucke, 2016).

Three critical insights emerge for clinical practice and future research. First, subclinical epileptiform activity affects up to half of AD patients and accelerates cognitive decline by 1.5-fold yet remains undetected without prolonged monitoring (Vossel et al., 2016a; Horvath et al., 2021). These finding mandates expanded use of ambulatory and overnight EEG in dementia evaluation, particularly when cognitive fluctuations or unexplained confusion are present. Second, distinct protein pathologies converge on network hyperexcitability through overlapping mechanisms including GABAergic interneuron dysfunction, glutamatergic excitotoxicity, and kinase dysregulation, creating potential for unified therapeutic approaches that target network function rather than individual molecular pathways (Palop and Mucke, 2016; Zott et al., 2019). Third, levetiracetam and related SV2A modulators offer not only seizure control but potential disease-modifying effects through reduction of network hypersynchrony, modulation of amyloid processing, and prevention of pathological protein spreading (Sanchez et al., 2012; Vossel et al., 2021).

The system epilepsy framework provides conceptual grounding for network-targeted interventions and supports the rationale for treating subclinical epileptiform activity to potentially modify disease course (Avanzini et al., 2012). Just as Rolandic epilepsy reflects hyperexcitability of the maturing sensorimotor system, dementia-associated epilepsy may reflect hyperexcitability of degenerating memory and cognitive networks. This paradigm shift transforms clinical approach from searching for focal epileptogenic zones toward understanding and modulating diffuse network dysfunction.

Critical knowledge gaps remain. Large-scale randomized controlled trials with epileptiform activity as a patient selection criterion and longitudinal cognitive outcomes as primary endpoints are needed to definitively establish whether antiseizure treatment modifies disease progression. Biomarkers that predict which patients will develop epileptiform activity or clinical seizures would enable targeted screening and early intervention. Head-to-head comparisons of different ASMs in dementia populations would inform optimal drug selection. Investigation of whether the system epilepsy characteristics of dementia-associated seizures predict different treatment responses than focal symptomatic epilepsies is warranted.

The integration of epilepsy management into dementia care represents an important evolution in clinical practice. By recognizing epileptiform activity as both a biomarker and therapeutic target, clinicians can offer patients interventions that may address not only seizure risk but potentially slow the inexorable cognitive decline that defines neurodegenerative dementias. This expanded perspective promises to improve outcomes for the growing population of individuals affected by these devastating conditions.
